# Enterovirus 75 Encephalitis in Children, Southern India

**DOI:** 10.3201/eid1611.100672

**Published:** 2010-11

**Authors:** Penny Lewthwaite, David Perera, Mong How Ooi, Anna Last, Ravi Kumar, Anita Desai, Ashia Begum, Vasanthapuram Ravi, M. Veera Shankar, Phaik Hooi Tio, Mary Jane Cardosa, Tom Solomon

**Affiliations:** Author affiliations: University of Liverpool, Liverpool, UK (P. Lewthwaite, M.H. Ooi, A. Last, T. Solomon);; Universiti Malaysia Sarawak, Sarawak, Malaysia (D. Perera, M.H. Ooi, P.H. Tio, M.J. Cardosa);; Sibu Hospital, Sibu, Sarawak (M.H. Ooi);; Vijayanagar Institute of Medical Sciences, Karnataka, India (R. Kumar, A. Begum, M. Veera Shankar);; National Institute of Mental Health and Neurological Sciences, Bangalore, India (R. Kumar, A. Desai, V. Ravi)

**Keywords:** Enterovirus 75, viruses, encephalitis, children, diagnostics, India, dispatch

## Abstract

Recent outbreaks of enterovirus in Southeast Asia emphasize difficulties in diagnosis of this infection. To address this issue, we report 5 (4.7%) children infected with enterovirus 75 among 106 children with acute encephalitis syndrome during 2005–2007 in southern India. Throat swab specimens may be useful for diagnosis of enterovirus 75 infection.

Sequences of enterovirus 75 (EV75) were first identified in cerebrospinal fluid (CSF), stool samples, and throat samples obtained during 1974–2000 in Ethiopia, Oman, Bangladesh, and the United States; the new enterovirus serotype was proposed in 2004 (*1*). Manifestations include upper respiratory tract infections and acute flaccid paralysis. In 2005–2006, EV75 was associated with aseptic meningitis in Spain (*2*,*3*). Enteroviruses have long been associated with encephalitis, but recent outbreaks of EV71 in Southeast Asia have highlighted the diagnostic difficulties that may be encountered (*4*–*6*). We report EV75 associated with encephalitis in India.

## The Study

We prospectively studied children <16 years of age who came to the Pediatric Department at the Vijayanagar Institute of Medical Sciences, Bellary, Karnataka, southern India, with acute encephalitis syndrome during October 2005–December 2007. Children with acute fever and symptoms with an onset <14 days before coming to the hospital, a neurologic illness, and >1 of the following signs (change in mental status including confusion, disorientation, coma, or inability to talk; new onset of seizures excluding simple febrile seizures; photophobia, headache, or meningitis) were recruited into the study. Children with laboratory-confirmed *Plasmodium falciparum* parasitemia or a history of neurologic conditions and those whose parents removed them from the hospital were excluded. The study protocol was reviewed and approved by the ethics committees of Vijayanagar Institute of Medical Sciences and the University of Liverpool, UK. Informed consent was obtained from parents or guardians.

For each child, a completed medical history was obtained and a detailed physical examination was performed. Samples were obtained for routine diagnostics. Rectal and throat samples were also collected after December 2005. Swabs were placed in sterile vials containing viral culture media (Dulbecco’s modified Eagle’s medium). All samples were frozen at –20°C, stored at –70°C, and transferred to diagnostic laboratories.

To detect enteroviruses, including EV75, in throat swab extracts, RNA was extracted by using the Chemagic Viral DNA/RNA kit (Chemagen AG, Baesweiler, Germany) and a Kingfisher mL Magnetic Extractor (Thermo Fisher Scientific Inc., Waltham, MA, USA). Pan-enterovirus reverse transcription–PCR (RT-PCR) was then performed by using primers specific for the 5′ untranslated region (5′-ATT GTC ACC ATA AGC AGC CA-3′ and 5′-CCT CCG GCC CCT GAA TGC GGC TAA T-3′); these primers produced a 154-bp product (*7*). Enteroviruses identified by pan-enterovirus RT-PCR were typed by nucleotide sequencing of the viral protein 1 (VP1) region (*8*,*9*).

To further type enteroviruses, phylogenetic analysis was performed on all nucleotide sequences (from this study and others obtained from GenBank) by using MEGA4 software (*10*) (www.megasoftware.net). The remaining swab transport medium was filtered, and 100 μL of filtrate was aliquoted onto each of 3 tissue culture plates containing rhabdomyosarcoma, Vero, and 293T cell lines for virus isolation. Cell lines were chosen to facilitate culturing of a range of viruses that may have been responsible for the clinical spectrum of illness seen.

Because CSF volume for virus isolation was limited to a 100-μL sample, this sample was placed on rhabdomyosarcoma cells for isolation and identification of enteroviruses. All CSF and serum samples were tested by using an immunoglobulin M capture ELISA to detect antibodies to Japanese encephalitis virus (JEV) and dengue virus, which circulate in the study region (*11*). If a sufficient amount of CSF remained, RNA was extracted by using the Viral RNA Mini Extraction Kit (QIAGEN, Hilden, Germany) according to the manufacturer’s instructions for pan-enterovirus RT-PCR, as described above and for a JEV RT-PCR (*7*,*12*). Plasma samples were also tested for chikungunya virus by using RT-PCR (*13*).

Of 243 children recruited into the study, 3 died before diagnostic samples could be obtained and 8 left the hospital against medical advice before samples were obtained. Among 232 children who satisfied the inclusion criteria, 166 CSF samples were obtained from 152 children. We also obtained 108 throat swabs specimens from 106 children and 19 rectal swab specimens from 18 children from the 171 children recruited after December 2005 when swab sampling began.

Virus isolates from throat swabs of 5 (4.7%) of 106 patients with a clinical signs of acute encephalitis syndrome were positive by pan-enterovirus RT-PCR for EV75. Sequencing indicated that VP1 regions of virus isolates obtained were similar to those of EV75. The 5 EV75-positive children were admitted to hospital on days 1–6 (median 4 days) of illness (Table). These children had a median age of 8 years (range 1.5–10 years), and 3 were girls. All 5 children had severe clinical disease (drowsiness, irritability, and reduced consciousness) at the time of admission, and 3 had seizures before admission.

**Table:** Characteristics and samples tested for 5 patients with confirmed enterovirus 75 infection, southern India*

Patient no.	Age, y/sex	Illness duration/length of hospital stay, d	Clinical signs†	Sample tested for EV75
1	6/F	6/7	5 d of fever and rigors, 3 d of headache, 2 d of neck pain and cough, and 1 d of photophobia. Drowsy and irritable with brisk reflexes on admission.	Throat swab and CSF
2	1.5/M	8/8	8 d of fever, rigors, coryzal symptoms, drowsiness, and mouth twitching; and 3 episodes of GTC seizures of 5–10 min duration. Still vacant and communication reduced at discharge.	Throat swab and CSF
3	10/F	3/5	3 d of fever, headache, and vomiting; 2 d of drowsiness at home; and 1 episode of GTC convulsion at home.	Throat swab
4	8/M	8/7	8 d of fever, headache, neck pain, cough, vomiting, and reduced speech and irritability. Irritable at discharge.	Throat swab and CSF
5	8/F	2/1	2 d of fever and 1 episode of GTC convulsion at home lasting 30 min.	Throat swab and CSF

*EV75, enterovirus 75; CSF, cerebrospinal fluid; GTC, generalized tonic clonic. †At admission to study.

Lumbar puncture was performed for 4 of the 5 children. Laboratory values were within reference ranges for 2 children, leukocyte counts were increased for 3 children, and results were not available for 1 child. The child for whom no lumbar puncture was performed had neurologic deterioration, meningism, and bilateral papilloedema; this child was treated empirically.

Sequencing showed that VP1 region homology for virus isolated in this study was similar to that observed for previously identified EV75 isolates (Figure). Sufficient CSF sample for pan-enterovirus RT-PCR was available for 162 samples from 162 patients, including 4 of the 5 EV75-positive patients; all samples were negative for JEV. These 162 samples were also negative for enteroviruses by pan-enterovirus RT-PCR (*7*,*12*). Serum samples from the 5 EV75-positive patients were negative for JEV and dengue virus by immunoglobulin M capture ELISA, for JEV by RT-PCR, and for chikungunya virus by RT-PCR. Eight patients showed positive results for chikungunya virus by RT-PCR, 48 showed positive results for JEV by ELISA, and 12 showed positive results for dengue virus by ELISA (*14*).

**Figure:**
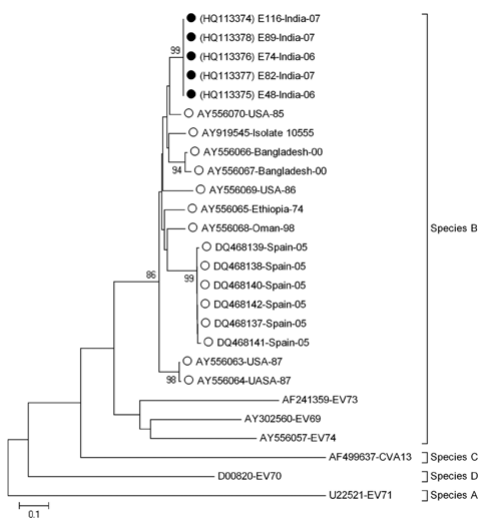
Phylogenetic analysis of viral protein 1 enterovirus 75 (EV75) nucleotide sequences. The tree was constructed by using the neighbor-joining method and the maximum-composite likelihood-substitution model. Significance of phylogenies was investigated by bootstrap analysis with 1,000 pseudoreplicate datasets. Bootstrap values >70% are indicated on the tree. Closed circles indicate isolates from India (this study) and open circles previously reported EV75 sequences. All EV75 sequences are named by using the conventional GenBank accession numbers (published sequences) or sample name (sequences from this study), country of origin, and year of isolation (where available). All prototype EV reference sequences are indicated by their GenBank accession numbers preceding serotype identification. Scale bar indicates nucleotide substitutions per site.

## Conclusions

Enteroviruses are a diverse group, and preferred samples for their diagnosis differ. Species B enteroviruses such as coxsackie virus A9 and Echo viruses B1–6 are usually readily isolated from CSF, unlike species A enteroviruses such as EV71. Although EV75 is a species B enterovirus, we were unable to isolate enterovirus from CSF from any of the patients, although throat swabs specimens were positive. This finding is similar to that for EV71, which has been isolated more frequently from throat swab specimens than from CSF or vesicles (*4*). In a study from Spain, EV75 was isolated from the CSF of 5 patients and nasopharyngeal swab specimens of 3 patients (*3*). However, throat swab specimens are rarely obtained in rural hospital settings with limited diagnostic facilities.

Although EV75 infection may have been a coincidental finding for the patients, we believe that this possibility is unlikely because results for other common causes of encephalitis were negative. Results of CSF testing were negative for other circulating viruses, including JEV, dengue virus, and herpes simplex virus, and patients also showed negative results for JEV and dengue virus by ELISA.

Our results illustrate the need to confirm diagnoses of EV75 in a range of specimens and by a range of laboratory investigations. This confirmation is required in India where encephalitis is often diagnosed clinically and recent outbreaks have been attributed to JEV. Laboratory diagnosis is hampered by single samples and high cost and low reliability of diagnostic tests currently available. Our study also shows that EV75 can cause encephalitis, in addition to aseptic meningitis and acute flaccid paralysis. Thus, as for other enteroviruses, throat swab specimens may be especially useful for diagnosis of infection with EV75.
